# Effects of osmotic pressure on the irreversible electroporation in giant lipid vesicles

**DOI:** 10.1371/journal.pone.0251690

**Published:** 2021-05-14

**Authors:** Malay Kumar Sarkar, Mohammad Abu Sayem Karal, Marzuk Ahmed, Md. Kabir Ahamed, Shareef Ahammed, Sabrina Sharmin, Sayed Ul Alam Shibly

**Affiliations:** 1 Department of Physics, Bangladesh University of Engineering and Technology, Dhaka, Bangladesh; 2 Department of Arts and Sciences, Ahsanullah University of Science and Technology, Dhaka, Bangladesh; 3 Department of Basic Science, Primeasia University, Dhaka, Bangladesh; Consiglio Nazionale delle Ricerche, ITALY

## Abstract

Irreversible electroporation (IRE) is a nonthermal tumor/cell ablation technique in which a series of high-voltage short pulses are used. As a new approach, we aimed to investigate the rupture of giant unilamellar vesicles (GUVs) using the IRE technique under different osmotic pressures (Π), and estimated the membrane tension due to Π. Two categories of GUVs were used in this study. One was prepared with a mixture of dioleoylphosphatidylglycerol (DOPG), dioleoylphosphatidylcholine (DOPC) and cholesterol (chol) for obtaining more biological relevance while other with a mixture of DOPG and DOPC, with specific molar ratios. We determined the rate constant (*k*_p_) of rupture of DOPG/DOPC/chol (46/39/15)-GUVs and DOPG/DOPC (40/60)-GUVs induced by constant electric tension (*σ*_c_) under different Π. The *σ*_c_ dependent *k*_p_ values were fitted with a theoretical equation, and the corresponding membrane tension (*σ*_oseq_) at swelling equilibrium under Π was estimated. The estimated membrane tension agreed well with the theoretical calculation within the experimental error. Interestingly, the values of *σ*_oseq_ were almost same for both types of synthesized GUVs under same osmotic pressure. We also examined the sucrose leakage, due to large osmotic pressure-induced pore formation, from the inside of DOPG/DOPC/chol(46/39/15)-GUVs. The estimated membrane tension due to large Π at which sucrose leaked out was very similar to the electric tension at which GUVs were ruptured without Π. We explained the *σ*_c_ and Π induced pore formation in the lipid membranes of GUVs.

## 1 Introduction

Lipid vesicles are closed and spherical structures formed by phospholipid bilayers which are used as model of biological cells [1]. When cells/vesicles are transferred into a hypotonic solution, the swelling of cells/vesicles occurs due to osmotic pressure (Π). The swelling induces a lateral tension in the membranes of cells/vesicles. Such membrane tension plays an important role in various physiological functions and mechanical properties of bio/lipid membranes [2–4]. Different types of responses such as an increase in volume, and leakage of internal contents have been investigated under Π using large unilamellar vesicles (LUVs) and giant unilamellar vesicles (GUVs) [5–11]. The elastic properties of membranes were measured by investigating the changes of volume in presence of Π using dynamic light scattering [5, 6]. Since the static and dynamic change in size and shape of a ‘single GUV’ can be visualized using the optical microscope, vesicles with sizes 10 μm or more have been using in various researches [12, 13]. When GUVs are transferred to a hypertonic solution, the shrinkage of GUVs occurs. The analysis of the time-dependent decrease in volume of each GUV provides the water permeability of lipid membranes [14, 15]. The main advantage to use the GUVs is that any change of a ‘single GUV’ can be measured independently, while not possible for the case of LUVs as it provides an ensemble average of many small nanometers sized vesicles. Therefore, the size of GUVs provides the opportunity to observe the phenomena occurring at the single individual vesicle.

Irreversible electroporation (IRE) is a cell/tumor ablation technique that uses direct current (DC) pulses of length 100 μs to 1 ms, electric field of magnitude hundreds to thousands of V/cm, and frequency of around 1.1 kHz (i.e, electrical pulses of width 200 μs are delivered once per 909 μs). The electric field induces lateral tension in the membranes of GUVs, resulting in the permanent permeabilization of the membranes of vesicles [16, 17]. If the lateral tension reaches a critical value, pore formation occurs in membranes and subsequently vesicles are ruptured. The permeabilization due to transient pores or transmembrane pores is used for various medical and bioengineering purposes [18–20]. The continuous trajectory of pore formation in vesicles under applied tension or electrical stress is described by a theoretical model. The waiting time for the formation of a pore follows a nonmonotonous function of lateral tension in which the waiting time drops from infinity at zero tension to a minimum at any other tensions. On contrary, electric tension causes the waiting time to decrease monotonously [21]. The results of molecular dynamic (MD) simulations show that the kinetics of pore formation follows a linear dependency of activation energy of prepore formation under the applied field [22]. The pore life cycle due to the electric field describes the pore opening, construction and closing. The time of pore opening depends strongly on the gradient of electric field across the membrane interface and that the pore closing time is weakly dependent on the pore-initiating electric field. The pore closing time is much higher than the pore opening time [23]. The formation of a pore starts with the creation of single-file-like water defects penetrating the membranes under electric tension [24]. The metastability of a small hydrophilic pore is investigated by MD simulations, which is due to the compensating of positive and negative curvature effects at the edge of a pore [25]. Therefore, the research on the electric filed-induced pore formation is rapidly growing due to its potential applications in biology, biotechnology and medicine [18, 26]. It has been reported that GUVs prepared by hybrid films of agarose and lipids exhibit altered mechanical properties [27]. The presence of agarose meshwork inside the GUVs prevents the creation of pores in response to electric pulses and keep the membrane highly permeable. Incorporation of membrane proteins and cytoskeleton network in GUVs further improve the understanding of these elements in electropermeabilization.

It is well reported that plasma membranes of mammalian cells contain an amount of 20–50% cholesterol [28], which is an important element of lipid rafts involves in signal transduction and endocytosis [29, 30]. Since IRE technique is used for the ablation of tumor/cancer cells, it is important to consider the GUVs prepared by lipids and cholesterol for obtaining biological relevance. The presence of cholesterol increases the value of breakdown potential. The greater stability of membranes with cholesterol results in an increase of critical pore radius [31]. Also, the rate of pore formation is much slower for cholesterol containing membranes subjected to the electric field due to the substantial increment of membrane cohesion [32]. An addition of cholesterol increases the electroporation threshold that often links to the rise of the stiffness of bilayer [33]. The effects of cholesterol on the electric tension-induced pore formation is investigated where the line tension of a pore increased with the increase of cholesterol in the membranes [34]. Therefore, it is very important to study the GUVs under osmotic pressure at a more physiologically relevant condition. Here, we investigated the influence of osmotic pressure on the cholesterol containing charged membranes of GUVs along with the cholesterol free charged membranes of GUVs in a buffer containing a physiological concentration of ions. We used GUVs comprising a mixture of three elements: DOPG, DOPC and cholesterol, and also two elements: DOPG and DOPC. The electrostatic interaction due to the surface charges of membranes [35, 36] of DOPG/DOPC/chol (46/39/15)-GUVs and DOPG/DOPC (40/60)-GUVs is comparable.

We investigated the constant electric tension (*σ*_c_)-induced rate constant (*k*_p_) of rupture of GUVs under different Π. From the analysis of these investigations, the lateral membrane tension (*σ*_oseq_) due to Π at swelling equilibrium is evaluated. The experimentally estimated value of membrane tension is compared with the value calculated theoretically. The values of membrane tension of DOPG/DOPC/chol (46/39/15)-GUVs and DOPG/DOPC (40/60)-GUVs at a particular Π is found almost same. We also investigated the large osmotic pressure-induced sucrose leakage from the inside of DOPG/DOPC/chol (46/39/15)-GUVs. The sucrose leakage occurred owing to the formation of pores in the membranes of vesicles. Finally, we explained the σ_c_ and Π induced pore formation in GUVs.

## 2 Materials and methods

### 2.1 Chemical and reagents

DOPG and DOPC lipids were purchased from Avanti Polar Lipids Inc. (Alabaster, AL). Cholesterol (i.e. chol, *C*_h_) was purchased from WAKO pharmaceuticals (Japan). Bovine serum albumin (BSA), Piperazine-1, 4-bis (2-ethanesulfonic acid) (PIPES), O,O′-Bis (2-aminoethyl) ethyleneglycol-*N*,*N*,*N*′,*N*′,-tetraacetic acid (EGTA) were purchased from Sigma-Aldrich (Germany).

### 2.2 Preparation of GUVs

DOPG/DOPC/chol (46/39/15)-GUVs (46/39/15 indicates molar ratio) and DOPG/DOPC (40/60)-GUVs were prepared in a buffer (10 mM PIPES, 150 mM NaCl, pH 7.0, 1 mM EGTA) containing 100 mM sucrose using the well-known natural swelling method [37, 38]. The surface charge density of DOPG/DOPC (40/60)-GUVs is $\Omega_{\mathrm{PG}}=\frac{eX}{a_{\mathrm{DOPG}}}$ = – 0.10 Cm^-2^ where, *X* (= 0.40) is the DOPG molar fraction in membranes, *e* is the electronic charge and *a*_DOPG_ (= 72.5 Å^2^/molecule) is the cross sectional area of DOPG [39]. After the addition of cholesterol, the membranes become condensed [40–43]. Hence, the value of *a*_DOPG_ is about 50 Å^2^/molecule for 15 mole% cholesterol [44, 45]. The cross sectional area of cholesterol, *a*_ch_, is about half of *a*_DOPG_ i.e., 33–38 Å^2^//molecule [41]. Based on these values the surface charge density of DOPG/DOPC/chol (46/39/15)-GUVs is $\Omega_{\mathrm{ch}}=\frac{eX}{a_{\mathrm{DOPG}}\left(1-C_{h}\right)+a_{\mathrm{ch}}C_{h}}$ = – 0.15 C/m^2^. Therefore, the surface charge density of cholesterol containing membrane is slightly higher than the cholesterol free membrane. For preparing DOPG/DOPC/chol (46/39/15)-GUVs, a mixture of 1 mM DOPG, DOPC and cholesterol of amount 200 μL and for DOPG/DOPC (40/60)-GUVs a mixture of 1 mM DOPG and DOPC of amount 200 μL were taken into a 4.5 mL glass vial. The mixture was dried with a mild flow of nitrogen gas for producing a thin and homogeneous film. After that the vial was placed in a vacuum desiccator for overnight. Next, an amount of 20 μL MilliQ water was added into the vial and pre-hydrated for 8 min at 45°C, and then the sample was incubated for 3 h at 37°C with the same buffer containing 100 mM sucrose. An amount of 280 μL buffer solution containing various glucose concentrations which were smaller than sucrose concentration was taken into a U-shaped silicon microchamber that was inserted into a slide glass. An amount of 20 μL unpurified GUVs suspension was provided into the solution of microchamber and then waited about 20–25 min for settle down the GUVs suspension into the bottom of microchamber and also for the swelling equilibrium of vesicles in the presence of Π. As we used a concentration gradient between sucrose and glucose solution, an osmotic gradient was created between the inside and outside of GUVs. To prevent the strong attraction between the GUVs and the glass surface of slide glass, the chamber was coated using a 0.10% (w/v) BSA dissolved in the same buffer containing same glucose concentrations. The GUVs were visualized using an inverted phase contrast microscope (Olympus IX-73, Japan) with a 20× objective at 25 ± 1°C (Tokai Hit, Japan). The images of GUVs were recorded using a charge-coupled device camera (Olympus DP22, Japan).

### 2.3 Method of applying constant electric tension on GUVs under osmotic pressure

As mentioned in the preparation section of DOPG/DOPC/chol (46/39/15)-GUVs the sucrose concentration inside the GUVs was 100 mM. We calculated the osmolarity (mOsm/L) from the concentration of sucrose and glucose in mM according to the reported paper [11]. The equations of osmolarity for sucrose and glucose in buffer are defined as *y* = 1.029*C* + 292.9 and *y* = 1.010*C* + 294.2, respectively, where *C* is the molar concentration of sucrose or glucose. According to the equation, 100 mM sucrose concentration is equal to 396 mOsm/L. However, at the time of pre-hydration an amount of 20 μL MilliQ water was used. Therefore, the osmolarity of the inside sucrose of GUVs ($C_{\mathrm{in}}^{0}$) was 396×1000/1020 = 388 mOsm/L.

For the application of electric field on DOPG/DOPC/chol (46/39/15)-GUVs, at first, an amount of 280 μL buffer solution containing different glucose concentrations such as 76.6 mM and 72.3 mM were taken into a microchamber. An amount of 20 μL unpurified GUVs suspension containing 98 mM (= 394 mOsm/L) sucrose solution was provided into the solution of microchamber, the glucose concentration in the outside of DOPG/DOPC/chol (46/39/15)-GUVs would be 78 and 74 mM respectively. Therefore, the osmolarities of glucose solution were *C*_out_ = 373 and 369 mOsm/L for 78 and 74 mM, respectively. The corresponding values of osmolarity difference between the inside and outside of GUVs were $\Delta C^{0}=C_{\mathrm{in}}^{0}-C_{\mathrm{out}}$ = 15 and 19 mOsm/L. Due to the gradient between sucrose and glucose concentrations, GUVs became swell as water molecules of glucose solution passed into the inside of GUVs through membranes. The osmotic gradient creates lateral tension in the membranes of DOPG/DOPC/chol (46/39/15)-GUVs. Similarly, the values of osmolarity of glucose solution were *C*_out_ = 375 and 371 mOsm/L for 80 and 76 mM, respectively for DOPG/DOPC (60/40)-GUVs. The corresponding values of osmolarity difference between the inside and outside of GUVs were $\Delta C^{0}=C_{\mathrm{in}}^{0}-C_{\mathrm{out}}$ = 13 and 17 mOsm/L for DOPG/DOPC (60/40)-GUVs.

Here, we describe the experimental technique on the electric field-induced rupture of vesicles in the presence of different Π. A MOSFET based IRE circuit was used for generating direct current (DC) pulses of frequency 1.1 kHz and pulse width 200 μs in which electric signal was controlled with a microcontroller. The detail of the method is described in our reported papers [38, 46]. The gold coated electrode was used for applying the electric field on GUVs.

**Fig 1:**
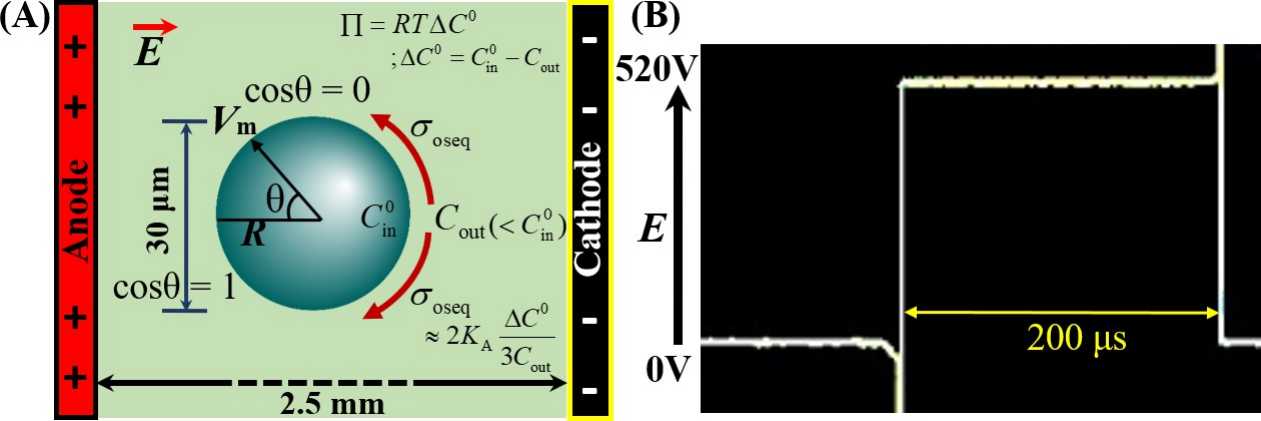
Illustration for applying the electric field in GUVs. (A) Schematic diagram of experimental set-up for the constant electric tension-induced rupture of GUVs under osmotic pressure. (B) An IRE signal of pulse width 200 μs.

To apply the constant electric tension on GUVs, initially the electric filed (*E*) was kept at around 320 V/cm and targeted a ‘single GUV’ located between the electrodes. Next, the value of *E* increased quickly (~7 s) to a definite level, and this *E* was kept constant until time 60 s. The experimental set-up for applying the electric field on GUVs under Π is illustrated in Fig 1.

Due to the application of *E* on a ‘single GUV’ with radius *R*, the internal and external free charges of buffer became polarize as the lipid membranes are impermeable to ions. The accumulation time of free charges is calculated by the Maxwell–Wagner equation [47]. According to the Maxwell stress tensor, accumulation of free charges induces a lateral electric tension in the membranes given by the following equation [47–49].

$$\sigma_{c}=\varepsilon_{m}\varepsilon_{0}(\frac{h}{2h_{e}^{2}})V_{m}^{2}$$ (1)

where, *V*_m_ (= 1.5*RE*) is the membrane potential by considering the membrane charging time *τ*_charg_ ≈ 0 [47, 50], *ε*_m_ (~ 4.5) is the membrane’s permittivity [50–52], *ε*_0_ is the free space permittivity, *h* (~ 4 nm) is the thickness of membrane [53] and *h*_e_ (~2.8 nm) is the membrane dielectric thickness [53, 54]. After simplification, Eq (1) can be written as follows [46].

$$\sigma_{c}=22.86R^{2}E^{2}[\mathrm{mN}/m]$$ (2)

We applied electric field, *E* = 320–560 V/cm in the experiment. For *R* = 10 μm and *E* = 553 V/cm, *V*_m_ = 0.83 V. The applied electric tension was considered constant as a fixed value of *E* was applied for the corresponding *R*. By choosing the appropriate value of *E*, various constant tensions were applied on GUVs.

### 2.4 Rate constant of tension-induced pore formation in GUVs under osmotic pressure

Under osmotic pressure, the electric field induced a total tension, *σ*_t_ = *σ*_c_ + *σ*_oseq_, in the membranes of GUVs, where *σ*_c_ is the tension due to electric field and *σ*_oseq_ is the tension due to Π at swelling equilibrium. Using the mean first passage time approach the rate constant (*k*_p_) of tension-induced pore formation in the membranes of GUVs is determined as follows [55]:

$$k_{p}=A_{F}\left(\sigma_{t}+B\right)\exp\left[-\frac{\pi \Gamma^{2}}{kT\left(\sigma_{t}+B\right)}\right]$$ (3)

where *A*_F_ is the pre-exponential factor, Γ is the line tension of membrane and *B* is the electrostatic interaction term due to charges of membrane. The theoretical Eq (3) was used to fit the experimental data on *k*_p_ vs. *σ*_t_ for both DOPG/DOPC/chol (46/39/15) and DOPG/DOPC (40/60)-GUVs.

### 2.5 Theoretical membrane tension of GUVs in presence of osmotic pressure

The theoretical aspect of membrane tension of GUVs in presence of Π is described in our previous paper [10]. Here, we present a brief description of the theory under Π. Let us consider a ‘single GUV’ of initial radius *r*_0_ and the initial osmolarity inside the GUV is $C_{\mathrm{in}}^{0}$. The unit of osmolarity (mOsm/L) is the same as mM (mmol/L or mol/m^3^). If the GUVs are transferred to a hypotonic solution of concentration *C*_out_ (mOsm/L), Π is induced in the GUV, resulting the radius of GUVs increases to Δ*r*_eq_ at swelling equilibrium. The osmolarity difference at initial condition between the inside and the outside of GUV becomes $\Delta C^{0}=C_{\mathrm{in}}^{0}-C_{\mathrm{out}}$ and then Π = *RT*Δ*C*^0^, where *R* is the gas constant and *T* is the absolute temperature. The membrane tension at swelling equilibrium is defined as follows [10]:

$$\sigma_{\mathrm{oseq}}=2K_{A}\frac{RT\Delta C^{0}/2}{\frac{2K_{A}}{r_{0}}+\frac{3RTC_{\mathrm{out}}}{2}-\frac{RT\Delta C^{0}}{2}}\approx 2K_{A}\frac{\Delta C^{0}}{3C_{\mathrm{out}}}$$ (4)

where, *K*_A_ is the area compressibility modulus of the membranes of GUVs. We reported the value of *K*_A_ = 141 ± 5 mN/m for DOPG/DOPC (40/60)-GUVs [12]. Using the same method (i.e., micropipette aspiration technique), we measured the value of *K*_A_ = 154 ± 4 mN/m for DOPG/DOPC/chol (46/39/15)-GUVs.

### 2.6 Large osmotic pressure-induced sucrose leakage from the inside of GUVs

For the measurement of large osmotic pressure-induced sucrose leakage from the inside of GUVs, at first, an amount of 280 μL buffer solution containing 61.57 mM (= 356 mOsm/L) glucose concentration was taken into a microchamber. An amount of 20 μL unpurified GUVs suspension containing 98 mM (= 394 mOsm/L) sucrose solution was provided into the solution of microchamber, the glucose concentration in the outside of DOPG/DOPC/chol (46/39/15)-GUVs would be 64 mM (= 359 mOsm/L). Therefore, the osmolarity difference between the inside and outside GUVs was $\Delta C^{0}=C_{\mathrm{in}}^{0}-C_{\mathrm{out}}$ = 388–359 = 29 mOsm/L. After 20–25 min of the application of osmotic gradient, we have taken several phase contrast images of GUVs suspension. We observe the sucrose leakage of GUVs as water molecules of glucose solution continuously passed through the membranes from the outside to the inside of GUVs. Due to the swelling of vesicles, GUVs leaked sucrose solution for releasing the membrane tension. The images of GUVs were recorded at 25 frames per second (fps) using a digital camera (Model: DP22, Olympus) connected to the microscope.

## 3 Results and analysis

### 3.1 Constant electric tension-induced stochastic rupture of DOPG/DOPC/chol (46/39/15)-GUVs under different osmotic pressures

**Fig 2:**
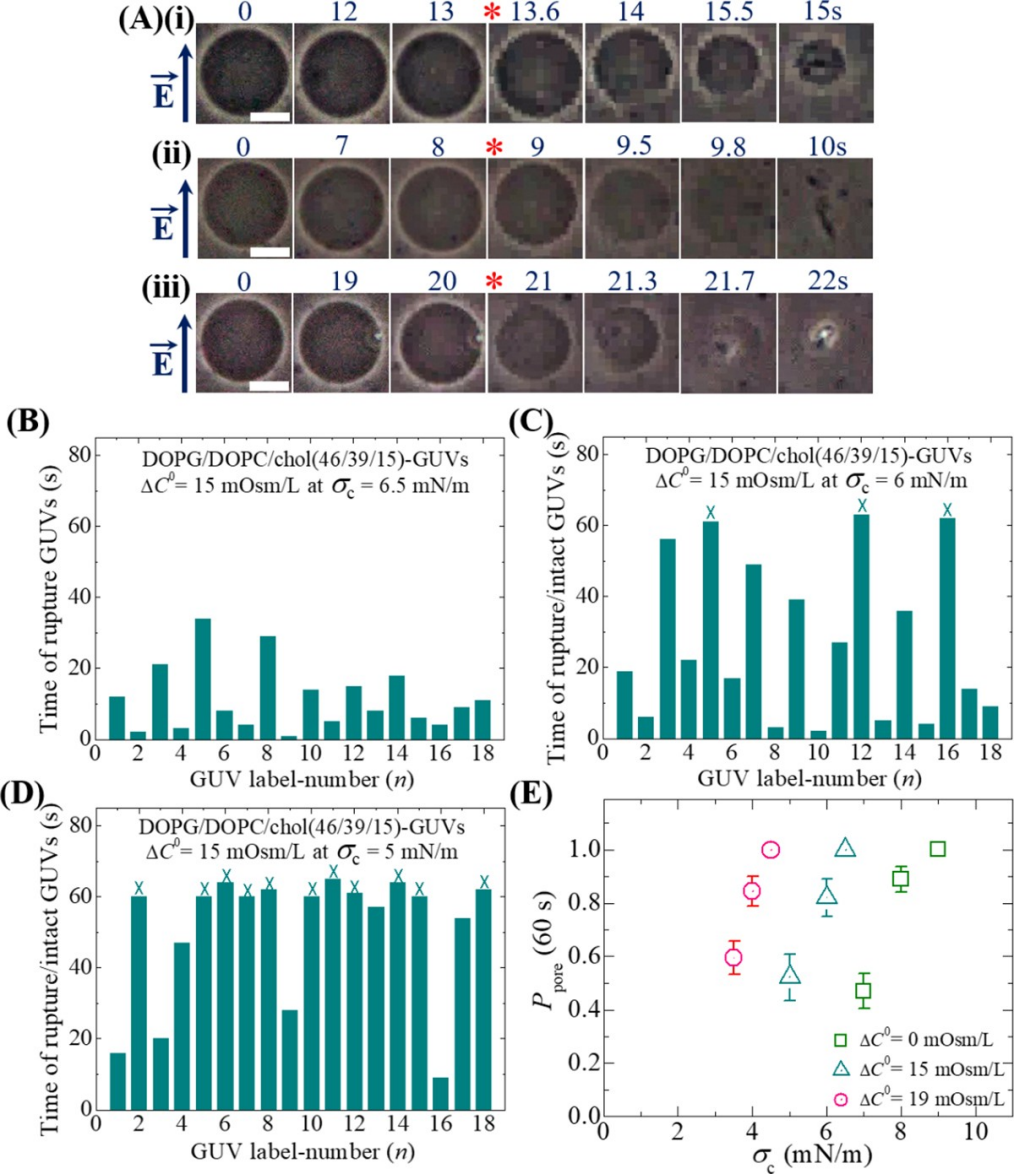
The influence of osmotic pressure on the constant electric tension-induced stochastic rupture of DOPG/DOPC/chol (46/39/15)-GUVs in a buffer containing physiological ions. (A) Phase contrast images of rupture of (i) first (ii) second and (iii) third ‘single DOPG/DOPC/chol (46/39/15)-GUV’ at tension, *σ*_c_ = 6.5 mN/m and the osmolarity difference between the inside and outside GUVs, Δ*C*^0^ = 15 mOsm/L. The field direction is shown with an arrow in the left side. The numbers above in each image indicate the time in seconds after applying of *σ*_c_ due to electric field. The white bar corresponds to a length of 15 μm. The time of stochastic rupture/intact in several single DOPG/DOPC/chol (46/39/15)-GUVs under Δ*C*^0^ = 15 mOsm/L at (B) *σ*_c_ = 6.5 mN/m (C) *σ*_c_ = 6.0 mN/m and (D) *σ*_c_ = 5.0 mN/m. The number of measured GUVs in B, C and D was 18. The bars in B, C, D represent ruptured and intact GUVs. The cross mark (×) above the bars in C, D indicates the intact GUVs until time 60 s. (E) The *σ*_c_ dependent *P*_pore_ (60 s) value for DOPG/DOPC/chol (46/39/15)-GUVs under Δ*C*^0^ = 0 (green open square), Δ*C*^0^ = 15 mOsm/L (cyan open triangle) and Δ*C*^0^ = 19 mOsm/L (pink open circle). Average values and standard deviations of *P*_pore_ (60 s) at *σ*_c_ were determined for 2–3 independent experiments, each with 15–24 GUVs, for each value of *σ*_c_.

We investigated the constant electric tension-induced rupture of GUVs at different osmotic pressures. To apply Π, DOPG/DOPC/chol (46/39/15)-GUVs suspension is transferred from an isotonic solution to a hypotonic solution. The initial osmolarity difference between the inside and outside of GUVs governs the values of Π. The value of osmolarity of sucrose solution was $C_{\mathrm{in}}^{0}$ = 388 mOsm/L. An amount of 20 μL GUVs suspension was provided with the same buffer of amount 280 μL containing 76.6 mM glucose concentration. Hence, the outside concentration of DOPG/DOPC/chol (46/39/15)-GUVs was 78 mM, which had osmolarity value *C*_out_ = 373 mOsm/L. The osmolarity difference between the inside sucrose and the outside glucose concentration of GUVs was Δ*C*^0^ = 388–373 = 15 mOsm/L. After providing the GUVs suspension into the microchamber, it was waited 20–25 min for homogeneous mixing of the solutions and also for the swelling equilibrium of GUVs in the presence of Π. At first, we applied a constant electric tension of value *σ*_c_ = 6.5 mN/m on a ‘single DOPG/DOPC/chol (46/39/15)-GUV’ for time 60 s. Before applying the electric tension due to the electric field, the GUV had a high contrast in an inverted phase contrast image as shown in Fig 2A(i) due to the difference in refractive indexes of sucrose and glucose solution (i.e., sucrose in the inside of GUVs and glucose in the outside of GUVs). During application of *σ*_c_, the spherical-shaped GUV was also intact until the time 13 s. At time 13.6 s, GUV initiated to rupture and at time 14 s GUV was ruptured completely (Fig 2A(i)). Therefore, the spherical structure of GUV was permanently disappeared due to the rupture of vesicle. In our several previous papers, we explained the rupture of GUVs in such a way that at first a nanopore is formed in the membranes whose radius rapidly increases to infinity, leading to complete rupture of GUVs [38, 46, 56]. The time of pore formation is defined as the time when the vesicles started to rupture.

We performed similar experiments for several ‘single DOPG/DOPC/chol (46/39/15)-GUVs’ (the number of examined GUVs, *n*_1_ = 15–24). Here, we present only three GUVs. Fig 2A(ii) and 2A(iii) represent the rupture of the 2^nd^ and 3^rd^ GUVs, in which the rupture occurs at time 9 s and 21 s, respectively. The starting time of rupture is indicated by asterisk mark (*). The rupture of several ‘single GUVs’ followed stochastic nature, which means that the rupture occurred at different times.

At Δ*C*^0^ = 15 mOsm/L, the stochastic rupture (i.e., pore formation) of several ‘single GUVs’ (*n*_1_ = 18) at tension *σ*_c_ = 6.5 mN/m is shown in Fig 2(B). The similar stochastic rupture of DOPG/DOPC/chol (46/39/15)-GUVs was also observed at *σ*_c_ = 6.0 and 5.0 mN/m (Fig 2C and 2D). The bars in the bar chart indicate the GUV label-number (*n*) until time 60 s (Fig 2B–2D). For the case of DOPG/DOPC/chol (46/39/15)-GUVs, rupture was occurred in all observed GUVs at *σ*_c_ = 6.5 mN/m, i.e., the probability of pore formation, *P*_pore_ (60 s) = 1.0. On contrary, the rupture was occurred in 15 GUVs out of 18 GUVs (i.e., *P*_pore_ (60 s) = 0.83) at *σ*_c_ = 6.0 mN/m, and at *σ*_c_ = 5.0 mN/m; the rupture was formed in 7 GUVs out of 18 GUVs (i.e., *P*_pore_ (60 s) = 0.39). The cross mark (×) above the bars (Fig 2C and 2D) indicates the intact GUVs until time 60 s.

These results clearly indicate that as the external tension decreased from 6.5 to 5.0 mN/m, the probability of rupture became smaller for DOPG/DOPC/chol (46/39/15)-GUVs. Fig 2(E) shows that *σ*_c_ dependent *P*_pore_ (60 s) value for DOPG/DOPC/chol (46/39/15)-GUVs at Δ*C*^0^ = 0 (green open square), Δ*C*^0^ = 15 mOsm/L (cyan open triangle) and Δ*C*^0^ = 19 mOsm/L (pink open circle). The experimental data of *σ*_c_ dependent *P*_pore_ (60 s) for DOPG/DOPC/chol (46/39/15)-GUVs under different osmolarity differences is provided in S1 Table of S1 File.

### 3.2 Constant electric tension induced rate constant of rupture in DOPG/DOPC/chol (46/39/15)-GUVs under different osmotic pressures

**Fig 3:**
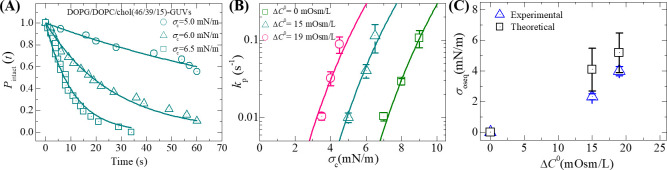
Constant electric tension-dependent rupture of DOPG/DOPC/chol (46/39/15)-GUVs under different osmotic pressures. (A) The time course of the fraction of intact DOPG/DOPC/chol (46/39/15)-GUVs at tensions *σ*_c_ = 6.5, 6.0 and 5.0 mN/m. The solid lines represent the best-fitted single exponential decay function of Eq (5). (B) The *σ*_c_ dependent *k*_p_ value for DOPG/DOPC/chol (46/39/15)-GUVs under the osmolarity difference between the inside and outside of GUVs, Δ*C*^0^ = 0 (green open square), Δ*C*^0^ = 15 mOsm/L (cyan open triangle) and Δ*C*^0^ = 19 mOsm/L (pink open circle). Average values with standard deviations of *k*_p_ at *σ*_c_ were determined for 2–3 independent experiments, each with 15–24 GUVs, for each value of *σ*_c_. The solid (green, cyan and pink) lines were the best fit theoretical curves corresponding to Eq (3) using line tension Γ = 12.9 pN, *B* = 2.14 mN/m and *A*_F_ = 8.4×10^5^ m^2^s^−1^ J^−1^. The cyan line and the pink line correspond to the theoretical Eq (3) using *σ*_t_ = *σ*_c_ + 2.3 mN/m and *σ*_t_ = *σ*_c_ + 4.0 mN/m, respectively. (C) The Δ*C*^0^ dependent membrane tension (*σ*_oseq_) (experimental and theoretical). Experimental values were determined by analyzing the constant electric tension-induced rupture of GUVs. Their mean values with standard deviations are shown.

To calculate the rate constant of rupture of GUVs induced by constant electric tension, we determined the time-dependent fraction of intact GUVs without rupture among all the examined GUVs, *P*_intact_ (*t*). It basically indicates the fraction of GUVs that are still intact after time ‘*t*’ and is defined as *P*_intact_ (*t*) = 1– *P*_pore_ (*t*) [57, 58]. Fig 3(A) shows the time course of *P*_intact_ (*t*) for DOPG/DOPC/chol (46/39/15)-GUVs at *σ*_c_ = 6.5 (square), 6.0 (triangle) and 5.0 mN/m (circle). As the value of *σ*_c_ decreases from 6.5 to 5.0 mN/m, the *P*_intact_ (*t*) with time is slower. The time-dependent *P*_intact_ (*t*) is well fitted by a single-exponential decay function (solid line in Fig 3A):

$$P_{\mathrm{intact}}(t)=\exp\left(-k_{p}t\right)$$ (5)

where *k*_p_ is the rate constant for rupture (i.e., pore formation) and *t* is the duration of constant electric tension applied to a GUV (tension is started at *t* = 0). The values of *k*_p_ were obtained 1.0×10^−1^, 4.0×10^−2^ and 0.9×10^−2^ s^-1^ for tensions 6.5, 6.0 and 5.0 mN/m, respectively. We performed the same experiments for 2–3 times at different tensions under the osmolarity difference between the inside and outside of GUVs, Δ*C*^0^ = 15 mOsm/L and calculated the average value with standard deviation of rate constant for each tension. Fig 3(B) shows the *σ*_c_ dependent *k*_p_ values for Δ*C*^0^ = 15 mOsm/L (cyan open triangle). Next, we investigated the constant electric tension-induced rupture of DOPG/DOPC/chol (46/39/15)-GUVs for a different Δ*C*^0^ using the same method. For this purpose, an amount of 20 μL GUVs suspension was provided into the microchamber containing the same buffer of amount 280 μL with 72.3 mM glucose concentration. Hence, the outside concentration of DOPG/DOPC/chol (46/39/15)-GUVs was 74 mM, which had osmolarity value *C*_out_ = 369 mOsm/L. The osmolarity difference between the inside sucrose and the outside glucose concentration of GUVs was Δ*C*^0^ = 388–369 = 19 mOsm/L. Using the similar procedure, we calculated the values of *k*_p_ under Δ*C*^0^ = 19 mOsm/L at tensions 4.5, 4.0 and 3.5 mN/m. The *σ*_c_ dependent *k*_p_ value for Δ*C*^0^ = 19 mOsm/L (pink open circle) is shown in Fig 3(B).

As a control experiment, the *σ*_c_ dependent *k*_p_ value at Δ*C*^0^ = 0 mOsm/L (green open square) for DOPG/DOPC/chol (46/39/15)-GUVs is also presented in Fig 3(B). The experimental data of *σ*_c_ dependent *k*_p_ for DOPG/DOPC/chol (46/39/15)-GUVs under different osmolarity differences is provided in S1 Table of S1 File. The Δ*C*^0^ dependent membrane tension for DOPG/DOPC/chol (46/39/15)-GUVs is shown in Fig 3(C). Tables 1 and 2 show the electric tension-dependent average rate constant of rupture of DOPG/DOPC/chol (46/39/15)-GUVs under Δ*C*^0^ = 15 and 19 mOsm/L, respectively.

**Table 1:** An estimation of membrane tension of DOPG/DOPC/chol (46/39/15)-GUVs for Δ*C*^0^ = 15 mOsm/L and *C*_out_ = 373 mOsm/L.

*σ*_c_ (mN/m) with Δ*C*^0^	Rate constant of rupture *k*_p_ (s^-1^)	*σ*_c_ (mN/m) with Δ*C*^0^ = 0 using Eq (3)	*σ*_osexp_ (mN/m) = *σ*_c_ (Δ*C*^0^ = 0)–*σ*_c_ (Δ*C*^0^)	Average value of experimental estimation *σ*_osexp_ (mN/m)	Theoretical estimation *σ*_osthe_ (mN/m)
5.0	(1.0 ± 0.1) × 10^−2^	7.3	2.3	2.3 ± 0.2	4.1 ± 1.4
6.0	(4.0 ± 0.8) × 10^−2^	8.2	2.2		
6.5	(1.1 ± 0.5) × 10^−1^	9.0	2.5		

**Table 2:** An estimation of membrane tension of DOPG/DOPC/chol (46/39/15)-GUVs for Δ*C*^0^ = 19 mOsm/L and *C*_out_ = 369 mOsm/L.

*σ*_c_ (mN/m) with Δ*C*^0^	Rate constant of rupture *k*_p_ (s^-1^)	*σ*_c_ (mN/m) with Δ*C*^0^ = 0 using Eq (3)	*σ*_osexp_ (mN/m) = *σ*_c_ (Δ*C*^0^ = 0)–*σ*_c_ (Δ*C*^0^)	Average value of experimental estimation *σ*_osexp_ (mN/m)	Theoretical estimation *σ*_osthe_ (mN/m)
3.5	(1.0 ± 0.1) × 10^−2^	7.2	3.7	4.0 ± 0.3	5.2 ± 1.3
4.0	(3.2 ± 0.7) × 10^−2^	8.0	4.0		
4.5	(0.9 ± 0.2) × 10^−1^	8.8	4.3		

### 3.3 Constant electric tension-induced rupture of DOPG/DOPC (40/60)-GUVs under different osmotic pressures

**Fig 4:**
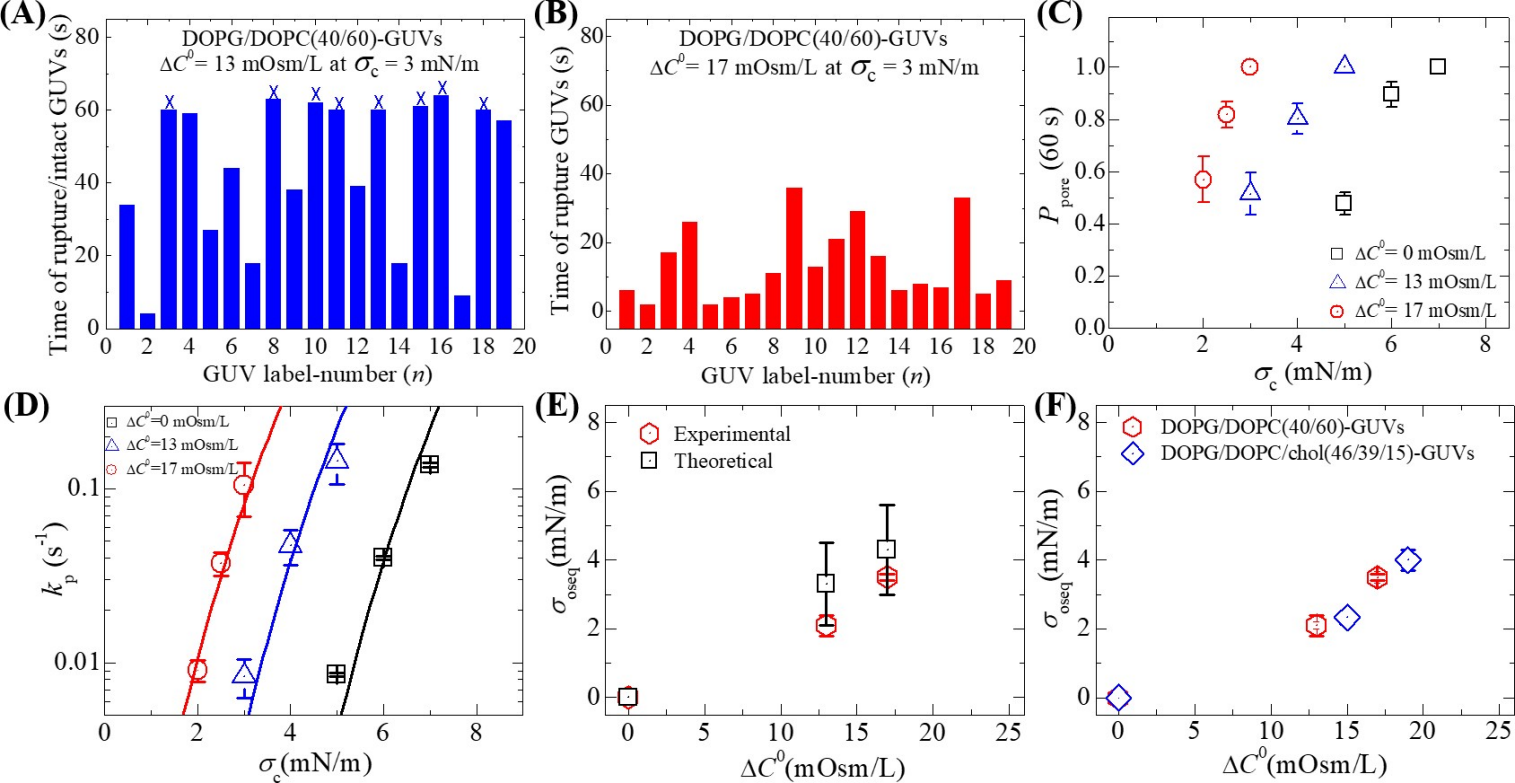
The time of stochastic rupture of several ‘single DOPG/DOPC (40/60)-GUVs’. (A) The osmolarity difference between the inside and outside of GUVs, Δ*C*^0^ = 13 mOsm/L (B) Δ*C*^0^ = 17 mOsm/L at *σ*_c_ = 3.0 mN/m. The number of measured GUVs in A and B was 19. The bars in A, B represent ruptured and intact GUVs. The cross mark (×) above the bars in A indicates the intact GUVs until time 60 s. (C) The *σ*_c_ dependent *P*_pore_ (60 s) value for DOPG/DOPC (40/60)-GUVs under Δ*C*^0^ = 0 (black open square), Δ*C*^0^ = 13 mOsm/L (blue open triangle) and Δ*C*^0^ = 17 mOsm/L (red open circle). (D) The *σ*_c_ dependent *k*_p_ value for DOPG/DOPC (40/60)-GUVs under Δ*C*^0^ = 0 (black open square), Δ*C*^0^ = 13 mOsm/L (blue open triangle) and Δ*C*^0^ = 17 mOsm/L (red open circle). Average values with standard deviations of *k*_p_ at *σ*_c_ were determined for 2–3 independent experiments, each with 15–24 GUVs, for each value of *σ*_c_. The solid (black, blue and red) lines were the best-fitted theoretical curves corresponding to Eq (3) using line tension Γ = 12.1 pN, *B* = 1.76 mN/m and *A*_F_ = 8.8×10^6^ m^2^s^−1^ J^−1^. The blue line and the red line correspond to the theoretical Eq (3) using *σ*_t_ = *σ*_c_ + 2.1 mN/m and *σ*_t_ = *σ*_c_ + 3.5 mN/m, respectively. (E) The Δ*C*^0^ dependent membrane tensions (*σ*_oseq_) (experimental and theoretical) at swelling equilibrium. Experimental values are determined by analyzing the constant electric tension-induced rupture of GUVs. Their mean values with standard deviations are shown. (F) Comparison of experimentally determined membrane tension for DOPG/DOPC (40/60) and DOPG/DOPC/chol (46/39/15)-GUVs under different Δ*C*^0^.

So far, we investigated the rupture of DOPG/DOPC/chol (46/39/15)-GUVs under different osmotic pressures. In the similar way, we investigated the constant electric tension-induced rupture at different Π for DOPG/DOPC (40/60)-GUVs. Fig 4(A) and 4(B) shows the stochastic rupture under Δ*C*^0^ = 13 and 17 mOsm/L, respectively at *σ*_c_ = 3.0 mN/m. The cross mark (×) above the bars in the bar chart indicates the intact GUVs until time 60 s. In these investigations, we calculated the values of *k*_p_ at various tensions under different Π. The *σ*_c_ dependent *P*_pore_ (60 s) value under Δ*C*^0^ = 13 mOsm/L (blue open triangle), Δ*C*^0^ = 17 mOsm/L (red open circle) and Δ*C*^0^ = 0 mOsm/L (black open square) is shown in Fig 4(C). Fig 4(D) shows the *σ*_c_ dependent *k*_p_ values under different Δ*C*^0^. The value of *k*_p_ increased with the increase of applied tension for a particular osmotic pressure. The experimental data of *σ*_c_ dependent *P*_pore_ (60 s) and *σ*_c_ dependent *k*_p_ for DOPG/DOPC (40/60)-GUVs under different osmolarity differences are provided in S2 Table of S1 File. The Δ*C*^0^ dependent membrane tension for DOPG/DOPC (40/60)-GUVs is shown in Fig 4(E). Fig 4(F) shows a comparison of experimentally determined membrane tension for DOPG/DOPC (40/60)-GUVs and DOPG/DOPC/chol (46/39/15)-GUVs. Tables 3 and 4 show the tension-dependent average rate constants of rupture for DOPG/DOPC (40/60)-GUVs under Δ*C*^0^ = 13 and 17 mOsm/L, respectively.

**Table 3:** An estimation of membrane tension of DOPG/DOPC (40/60)-GUVs for Δ*C*^0^ = 13 mOsm/L and *C*_out_ = 375 mOsm/L.

*σ*_c_ (mN/m) with Δ*C*^0^	Rate constant of rupture *k*_p_ (s^-1^)	*σ*_c_ (mN/m) with Δ*C*^0^ = 0 using Eq (3)	*σ*_osexp_ (mN/m) = *σ*_c_ (Δ*C*^0^ = 0)–*σ*_c_ (Δ*C*^0^)	Average value of experimental estimation *σ*_osexp_ (mN/m)	Theoretical estimation *σ*_osthe_ (mN/m)
3.0	(0.8 ± 0.2) × 10^−2^	5.3	2.3	2.1 ± 0.3	3.3 ± 1.2
4.0	(4.7 ± 1.1) × 10^−2^	6.1	2.1		
5.0	(1.4 ± 0.4) × 10^−1^	6.8	1.8		

**Table 4:** An estimation of membrane tension of DOPG/DOPC (40/60)-GUVs for Δ*C*^0^ = 17 mOsm/L and *C*_out_ = 371 mOsm/L.

*σ*_c_ (mN/m) with Δ*C*^0^	Rate constant of rupture *k*_p_ (s^-1^)	*σ*_c_ (mN/m) with Δ*C*^0^ = 0 using Eq (3)	*σ*_osexp_ (mN/m) = *σ*_c_ (Δ*C*^0^ = 0)–*σ*_c_ (Δ*C*^0^)	Average value of experimental estimation *σ*_osexp_ (mN/m)	Theoretical estimation *σ*_osthe_ (mN/m)
2.0	(0.9 ± 0.1) × 10^−2^	5.4	3.4	3.5 ± 0.1	4.3 ± 2.3
2.5	(3.7 ± 0.5) × 10^−2^	6.0	3.5		
3.0	(1.1 ± 0.5) × 10^−1^	6.6	3.6		

### 3.4 Analysis of constant electric tension-induced rupture of GUVs under different osmotic pressures

Now we analyze the results of Fig 3(B) for estimating the membrane tension (*σ*_oseq_) induced by osmotic pressure at swelling equilibrium. The total membrane tension, *σ*_t_ = *σ*_c_ + *σ*_oseq_, where the tension *σ*_c_ was due to the external electric field and the tension *σ*_oseq_ was due to Π. The rate constant of rupture was determined for *σ*_t_. Hence, the shifting of the *k*_p_ vs. *σ*_c_ curve from the right to left side under Δ*C*^0^ corresponds to *σ*_oseq_ (Fig 3B). The value of *σ*_oseq_ can be estimated experimentally by subtracting the value of *σ*_c_ for the GUVs under Π to induce a particular value of *k*_p_ from the value of *σ*_c_ for the GUVs under Δ*C*^0^ = 0 to induce the same *k*_p_ value (if *σ*_oseq_ = 0, *σ*_t_ = *σ*_c_). The experimental data on *k*_p_ vs. *σ*_c_ for DOPG/DOPC/chol (46/39/15)-GUVs under Δ*C*^0^ = 0 was fitted to Eq (3) (green square in Fig 3B). An example is presented here. For Δ*C*^0^ = 15 mOsm/L, the experimental value of *k*_p_ = 1.1 × 10^−1^ s^−1^ at *σ*_c_ = 6.5 mN/m, and *σ*_c_ under Δ*C*^0^ = 0, which induces the same *k*_p_ value at 9.0 mN/m according to Eq (3). By subtracting 6.5 mN/m from 9.0 mN/m, the experimental membrane tension due to Π at swelling equilibrium, *σ*_osexp_ = 2.5 mN/m is obtained. On the other hand, the theoretical estimation of membrane tension can be obtained using Eq (4). At Δ*C*^0^ = 15 mOsm/L, *σ*_osthe_ = 4.1 mN/m. We also determined *σ*_oseq_ for other values of *k*_p_ (Table 1). The average value with standard deviation of *σ*_oseq_ for 3 different *σ*_c_ values was 2.3 ± 0.2 mN/m for DOPG/DOPC/chol (46/39/15)-GUVs. Hence, at Δ*C*^0^ = 15 mOsm/L, *σ*_t_ = *σ*_c_ + 2.3 mN/m. Using the relation *σ*_t_ = *σ*_c_ + 2.3 mN/m and the same parameter as those used for Δ*C*^0^ = 0, we obtained the theoretical curve (Eq 3) for *k*_p_ as a function of *σ*_t_ (Fig 3B, cyan line), which fitted well to the experimental data.

Next, we analyzed the results for Δ*C*^0^ = 19 mOsm/L using the same method for DOPG/DOPC/chol (46/39/15)-GUVs. The average value of *σ*_oseq_ was 4.0 ± 0.3 mN/m for DOPG/DOPC/chol (46/39/15)-GUVs under Δ*C*^0^ = 19 mOsm/L (Table 2). Using this value, we obtained the theoretical curve for *k*_p_ as a function of *σ*_t_ (Fig 3B, pink line). Fig 3(C) shows that the experimental *σ*_oseq_ increases with an increase in Δ*C*^0^. The Δ*C*^0^ dependent experimentally estimated membrane tension and theoretical tension are provided in Fig 3(C). The experimental data on *σ*_oseq_ corresponds to the theoretical tension.

The similar procedure was followed for the experimental estimation of *σ*_oseq_ for DOPG/DOPC (40/60)-GUVs. Using the relation *σ*_t_ = *σ*_c_ + 2.1 mN/m and the same parameter as those used for Δ*C*^0^ = 0, we obtained the theoretical curve for *k*_p_ as a function of *σ*_t_ using Eq (3) (Fig 4D, blue line) under Δ*C*^0^ = 13 mOsm/L, which fitted well to the experimental data. Similarly, using the relation *σ*_t_ = *σ*_c_ + 3.5 mN/m and the same parameter as those used for Δ*C*^0^ = 0, we obtained the theoretical curve for *k*_p_ as a function of *σ*_t_ using Eq (3) (Fig 4D, red line) under Δ*C*^0^ = 17 mOsm/L, which fitted well to the experimental data. Therefore, the mean values of *σ*_oseq_ were obtained 2.1 ± 0.3 mN/m and 3.5 ± 0.1 mN/m for DOPG/DOPC (40/60)-GUVs under Δ*C*^0^ = 13 and 17 mOsm/L, respectively. Tables 3 and 4 show the experimental and theoretical estimation of membrane tension for DOPG/DOPC (40/60)-GUVs under Δ*C*^0^ = 13 and 17 mOsm/L, respectively.

We have determined the values of *σ*_oseq_ theoretically using Eq (4) for DOPG/DOPC/chol (46/39/15)-GUVs and DOPG/DOPC (40/60)-GUVs under different Π. The theoretical estimation of *σ*_oseq_ for DOPG/DOPC/chol (46/39/15)-GUVs under Δ*C*^0^ = 15 and 19 mOsm/L is provided in Tables 1 and 2, respectively. Similarly, the theoretical estimation of *σ*_oseq_ for DOPG/DOPC (40/60)-GUVs under Δ*C*^0^ = 13 and 17 mOsm/L is provided in Tables 3 and 4, respectively. By the comparison of experimental and theoretical values of *σ*_oseq_ shown in Tables 1–4, it has been considered that these values agree with each other within the experimental error. The error of theoretically estimated *σ*_oseq_ was calculated based on the errors of Δ*C*^0^, $C_{\mathrm{in}}^{0}$, *C*_out_, and *K*_A_. The error of osmotic pressure for the solutions of sucrose and glucose was ± 2.8 mOsm/kg. The relative errors in the osmolarity values of *C*_out_, $C_{\mathrm{in}}^{0}$, Δ*C*^0^, and *K*_A_ for Δ*C*^0^ = 13 mOsm/L were estimated 0.0072, 0.0095, 0.43, and 0.035, respectively. The values of Δ*C*^0^ with errors for DOPG/DOPC/chol (46/39/15)-GUVs were Δ*C*^0^ = 15 ± 4 mOsm/L, and Δ*C*^0^ = 19 ± 4 mOsm/L whereas for DOPG/DOPC (40/60)-GUVs they were Δ*C*^0^ = 13 ± 4 mOsm/L, and Δ*C*^0^ = 17 ± 4 mOsm/L.

### 3.5 Comparison of the results of IRE technique with micropipette technique

**Fig 5:**
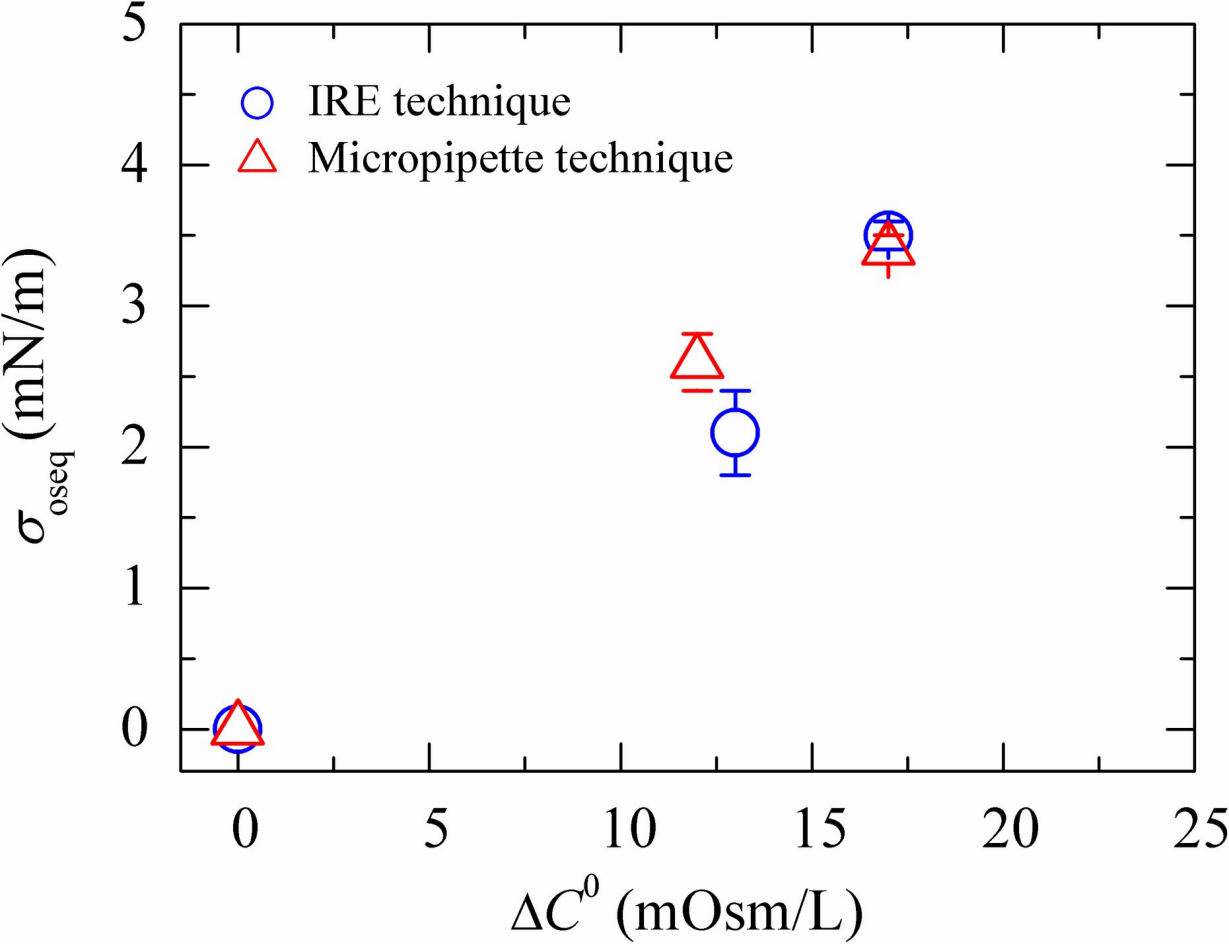
Comparison of the experimentally estimated membrane tension for DOPG/DOPC (40/60)-GUVs using the IRE technique and the micropipette technique. The data on the membrane tension using the micropipette technique was taken from [11].

To compare the electric tension-induced rupture of DOPG/DOPC(40/60)-GUVs to that of mechanical tension-induced rupture of the similar vesicles, it is important to show the results of the estimated membrane tension at different osmotic pressures. The procedure to apply the tension into the vesicles in two different techniques is totally different. In the IRE technique, the tension was applied using the Eq (1) whereas in the micropipette technique, tension was applied using an external force. In the latter case, the induced tension was calculated by the formula, *σ* = Δ*Pd*/4(1−*d*/*D*), where Δ*P* is the difference in pressure between the exterior and the interior of a micropipette, *d* is the inner diameter of the micropipette and *D* is the diameter of the spherical part of the GUV exterior to the micropipette. Fig 5 shows a comparison between the results of the IRE technique and the micropipette aspiration technique for DOPG/DOPC (40/60)-GUVs. The experimentally estimated membrane tension at different osmotic pressures for both the techniques provided similar values within the experimental error.

### 3.6 Large osmotic pressure-induced leakage of sucrose from GUVs

**Fig 6:**
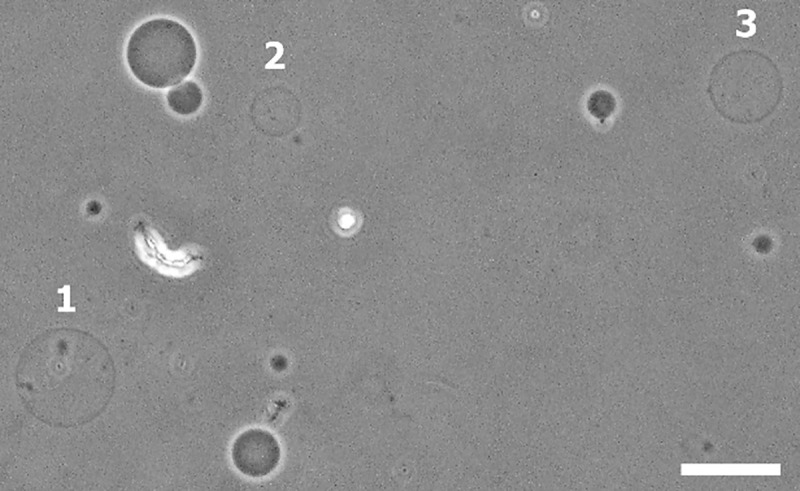
Phase contrast microscopic image of DOPG/DOPC/chol (46/39/15)-GUVs under the influence of the osmolarity difference between the inside and outside of GUVs, Δ*C*^0^ = 29 mOsm/L. Large osmotic pressure-induced leakage of sucrose due to the pore formation in the membranes of GUVs. The GUVs labeled by 1, 2 and 3 leaked out sucrose from their inside. The scale bar corresponds to a length of 50 μm.

Here, we investigated the large osmotic pressure-induced sucrose leakage from the inside of DOPG/DOPC/chol (46/39/15)-GUVs. In that case, the concentration gradient was Δ*C*^0^ = 388–359 = 29 mOsm/L. The images of GUVs were taken after 20–25 min of the addition of GUVs suspension into the glucose solution. A phase-contrast image of GUVs under Π is shown in Fig 6. In the presence of Δ*C*^0^, several GUVs labeled by 1, 2 and 3 leaked out sucrose. The sucrose leakage was confirmed by observing the contrast between the inside and outside of GUVs. It is to be noted that a few GUVs were still intact (no sucrose leakage occurred) under this osmolarity difference. The leakage of sucrose can be explained by the way that at first, GUVs were swelled as water molecules passed through the membranes from the outside of vesicles, and then, pores were formed in the membranes of GUVs for releasing the membrane tension in presence of Π. The same result was also obtained for several independent experiments (the number of independent experiments was 10). The fraction of GUVs in which sucrose leakage occurred during the first 20–25 min among all the examined GUVs was 0.55 ± 0.04 at Δ*C*^0^ = 29 mOsm/L. The membrane tension at Δ*C*^0^ = 29 mOsm/L was calculated theoretically using Eq (4) which was 8.0 mN/m.

## 4 Discussion

**Fig 7:**
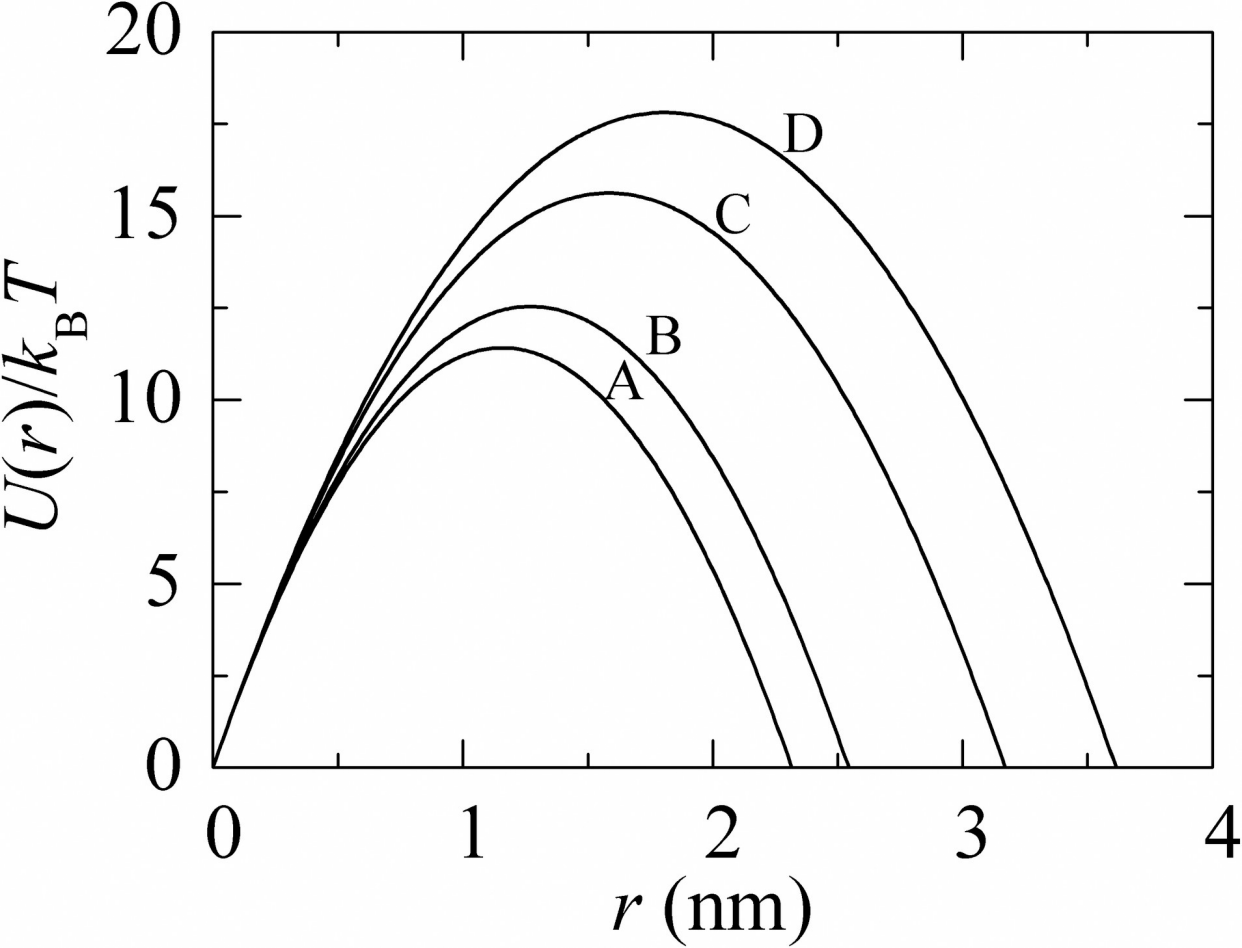
The prepore radius dependent free-energy profile of a prepore of DOPG/DOPC/chol (46/39/15)-GUVs under tension. (A) 9.0 mN/m (B) 8.0 mN/m (C) 6.0 mN/m and (D) 5.0 mN/m. *U*(*r*, *σ*_t_) was calculated according to Eq (6) using Γ = 12.9 pN.

We investigated the constant electric tension (*σ*_c_)-induced rupture of GUVs under different osmotic pressures, and then estimated the membrane tension (*σ*_oseq_) due to Π at swelling equilibrium from the analysis of results. If *σ*_oseq_ is greater than a critical value, the pore formation occurred in the membranes of GUVs. It is generally considered that the lipid membrane is an ensemble of lipid molecules in which local thermal fluctuation in the lateral density is always existing. The region where the lateral density is lower than their regular one is defined as a local density rarefaction or prepore of radius *r* [59, 60]. Under electric field and osmotic pressure, the total membrane tension *σ*_t_ = *σ*_c_ + *σ*_oseq_ is induced in the membrane. It is to be noted that if Π = 0, *σ*_t_ = *σ*_oseq_. If the size of such a rarefaction crosses a critical radius (*r*_c_), the region converts into a prepore of radius, *r*. If r < *r*_c_ the prepore closes rapidly, and if *r* ≥ *r*_c_ the prepore converts into a transmembrane pore. If *r* goes to infinite within a very short time (~1 s), GUVs become ruptured. The free energy of a prepore *U*(*r*, *σ*_t_) can be expressed as [38, 56, 57] *U*(*r*,*σ*_t_) = 2*πr*Γ−*πr*^2^*σ*_t_, where Γ is the free energy per unit length of a prepore (i.e., line tension) that is favoring the closure of a prepore. A similar equation of a prepore free energy for IRE technique was used in our previous paper [38] where toroidal structure of a prepore was considered [61, 62]. For DOPG/DOPC/chol (46/39/15)-GUVs and DOPG/DOPC (40/60)-GUVs, the free energy of a prepore can be written as,

$$U\left(r,\sigma_{t}\right)=2\pi \Gamma r-\pi r^{2}(\sigma_{t}+B)$$ (6)

where *B* is the electrostatic interaction term due to charged membranes [35, 56]. At the critical radius of a prepore, $r_{c}=\frac{\Gamma }{\sigma_{t}+B}$, the energy barrier of a prepore free energy is expressed as $U_{b}(r,\sigma_{t})=\frac{\pi \Gamma^{2}}{\sigma_{t}+B}$. The value of *B* ≈ 2.14 mN/m for DOPG/DOPC/chol (46/39/15)-GUVs and *B* ≈ 1.76 mN/m for DOPG/DOPC (40/60)-GUVs. Fig 7 shows an example of a prepore free energy for DOPG/DOPC/chol (46/39/15)-GUVs.

The time-dependent *P*_intact_ (*t*) data was fitted with a single-exponential decay function (Fig 3A), which suggested that the rupture can be considered as an irreversible two-state transition [56, 58]. From the fitted curve, we calculated the values of *k*_p_, which increased with *σ*_c_. On the other hand, the value of activation energy was determined by investigating the constant mechanical tension-induced pore formation [56, 58]. The tension-dependent activation energy clearly indicated that the classical theory of pore formation was correct [56, 58]. On the basis of classical theory, we explained the *σ*_c_ induced rupture of GUVs under different Π.

It is necessary to judge whether the values of *σ*_oseq_ and *σ*_c_ have the same effect for generating the lateral tension on the membranes or not. In the constant electric tension-induced rupture of GUVs (Figs 2–4), both *σ*_oseq_ and *σ*_c_ were applied to the GUVs, and hence the total membrane tension of vesicles was *σ*_t_ = *σ*_c_ + *σ*_oseq_. In the absence of Π, the values of *k*_p_ were obtained for the tensions 7.0, 8.0 and 9.0 mN/m for DOPG/DOPC/chol (46/39/15)-GUVs. When we applied such an osmotic gradient in GUVs which had a critical value of about 7 mN/m (according to Eq 4), rupture of GUVs occurred. In this regard, we investigated the sucrose leakage from the inside of GUVs under large Π, in which it was used only *σ*_oseq_ (i.e., *σ*_t_ = *σ*_oseq_). In the sucrose leakage experiment, we applied a value of *σ*_oseq_ = 8.0 mN/m for DOPG/DOPC/chol (46/39/15)-GUVs. Such investigation confirms the validity of the additive nature of *σ*_c_ and *σ*_oseq_ (i.e., *σ*_t_ = *σ*_c_ + *σ*_oseq_). If we compared the values of *σ*_t_ = *σ*_c_ (green open square in Fig 3B) and those of *σ*_t_ = *σ*_c_ + 2.3 (cyan open triangle in Fig 3B) and *σ*_t_ = *σ*_c_ + 4.0 (pink open circle in Fig 3B) for DOPG/DOPC/chol (46/39/15)-GUVs, it agreed with the experimental value within the error. The average values of membrane tensions were obtained 2.3 ± 0.2 and 4.0 ± 0.3 mN/m at Δ*C*^0^ = 15 and 19 mOsm/L, respectively for DOPG/DOPC/chol (46/39/15)-GUVs. To confirm the validity of the additivity of tensions such as *σ*_t_ = *σ*_c_ + *σ*_oseq_, we used it for the case of DOPG/DOPC (40/60)-GUVs under different Π as shown in Fig 4(D). In this case, the value of *σ*_t_ = *σ*_c_ (black open square in Fig 4D) at Δ*C*^0^ = 0 supported the tensions *σ*_t_ = *σ*_c_ + 2.1 (blue open triangle in Fig 4D) at Δ*C*^0^ = 13 mOsm/L and *σ*_t_ = *σ*_c_ + 3.5 (red open circle in Fig 4D) at Δ*C*^0^ = 17 mOsm/L for DOPG/DOPC (40/60)-GUVs. The average values of membrane tensions were obtained 2.1 ± 0.3 and 3.5 ± 0.1 mN/m at Δ*C*^0^ = 13 and 17 mOsm/L, respectively for DOPG/DOPC (40/60)-GUVs. These experimental estimations of membrane tension agreed with the theoretical value within the experimental error. The experimentally estimated membrane tension of DOPG/DOPC/chol (46/39/15)-GUVs and DOPG/DOPC (40/60)-GUVs for same Π was almost same as the values of area compressibility modulus for both membranes were very similar (Fig 4F). Such an additivity nature of tensions was also used when GUVs were induced by mechanical tension in the micropipette aspiration technique under different osmotic pressures [10, 11].

We can reasonably consider that *σ*_oseq_ induced leakage of sucrose due to the pore formation in membranes of GUVs occurred in a manner similar to *σ*_c_ induced rupture of GUVs. However, one important thing has to be discussed here. The electric field-induced rupture of vesicles did not follow the similar fashion of *σ*_oseq_ induced sucrose leakage, although in both cases pore formation was occurred in the membranes. In the case of *σ*_c_ induced rupture, at first a nanopore was formed in the membranes and its radius became infinity within a very short time in the presence of electric field, and therefore vesicle was ruptured. On the other hand, in the case of *σ*_oseq_ induced sucrose leakage, nanopores might not go to infinity within a very short time, and therefore, the vesicles became intact and spherical. Such intact with undetectable breaks of GUVs were also observed in the nanoparticles and antimicrobial peptide induced pore formation in the lipid membranes of GUVs [12, 63]. The estimated value of the critical radius of pore was 1.2 to 1.8 nm (as shown in Fig 7), which was very similar to that obtained by other investigations [10, 11].

During thermal fluctuation of membranes, if the radius of a prepore reaches to critical radius, transmembrane pore is formed, and hence causes a rapid leakage of sucrose from the inside of GUVs due to the Laplace pressure. Therefore, the internal content of GUVs leaked out (Fig 6), and consequently, the membrane tension vanished. It was reported experimentally the dynamics of closing of a large pore created by tension [64–66] and the theories of the evolution of a pore induced by tension explained well the results of closing of large pores [67, 68]. Hence, the growing of large Π-induced pore can be explained by these theories although it could not give any quantitative information for the closing of pore in this investigation. As the sucrose leakage occurred from the inside of GUVs, the phase contrast image of the leaked GUVs was different to that observed of the intact GUVs (Fig 6). The inside intensity of leaked GUVs labeled by 1, 2 and 3 (Fig 6) was less than that of intact GUVs. The similar intensity difference was also observed in nanoparticles-induced pore formation in GUVs [63]. Therefore, it was easy to differentiate the leaked GUVs from intact GUVs after large osmotic pressure-induced pore formation.

These investigations clearly indicated that the cell-size lipid vesicles such as GUVs became weak in the presence of Π. The formation of pore in plasma membranes of cells causes cell death, yet outside solute concentrations of cells change easily. To prevent Π induced cell death, cells modify their structure by incorporating mechanosensitive channels [4] into their plasma membranes during the development of life. The mechanosensitive channels open when membranes are stretched by Π [2, 3], and hence, the possibility of cell death became less in the presence of Π.

## 5 Conclusions

We investigated the effects of electric field-induced rate constant of rupture of DOPG/DOPC/chol (46/39/15)-GUVs and DOPG/DOPC (40/60)-GUVs under different osmotic pressure. Theoretical equation was fitted to the tension dependent rate constant of rupture of GUVs in the presence of different Π. The membrane tension was determined experimentally at swelling equilibrium under different Π using electroporation technique, which is a new approach. The estimated value of membrane tension agreed with the theoretical calculation. In the absence of external tension, a large value of osmotic pressure induced sucrose leakage from the inside of DOPG/DOPC/chol (46/39/15)-GUVs due to pore formation in the membranes of vesicles. The tension due to osmotic pressure and electric field determines the electric tension-induced rate constant of rupture of GUVs. The rupture was explained based on the classical theory of tension-induced pore formation in vesicles. These investigations provided quantitative and valuable information on membrane tension under different Π, which was important for research of the effects of Π on the activities of membrane proteins and membrane active peptides.

## Supporting information

S1 File (DOC)
