# Supplementary material for: Effects of osmotic pressure on the irreversible electroporation in giant lipid vesicles
Source: PLoS One. 2021 May 14;16(5):e0251690. doi: 10.1371/journal.pone.0251690 (PMC8121316; doi:10.1371/journal.pone.0251690)
Supplement: S1 File — (DOC) [file pone.0251690.s001.doc]

**S1 File**

**Effects of osmotic pressure on the irreversible electroporation in giant lipid vesicles**

Malay Kumar Sarkar, Mohammad Abu Sayem Karal, Marzuk Ahmed, Md. Kabir Ahamed, Shareef Ahammed, Sabrina Sharmin and Sayed Ul Alam Shibly

**S1 Table. The tension dependent probability of pore formation and the rate constant of rupture of DOPG/DOPC/chol (46/39/15)-GUVs under different osmolarities.**

| Electric tension  *σ*_c_ (mN/m) | Osmolarity difference Δ*C*^0^ (mOsm/L) | Probability of pore formation  *P*_pore_ (60 s) | Rate constant of rupture  *k*_p_ (s^-1^) |
| --- | --- | --- | --- |
| 7.0 | 0 | 0.47 ± 0.07 | (1.0 ± 0.1)×10^-2^ |
| 8.0 |  | 0.89 ± 0.05 | (2.9 ± 0.3)×10^-2^ |
| 9.0 |  | 1.0 ± 0.00 | (1.1 ± 0.3)×10^-1^ |
| 5.0 | 15 | 0.52 ± 0.09 | (1.0 ± 0.1)×10^-2^ |
| 6.0 |  | 0.82 ± 0.07 | (4.0 ± 0.8)×10^-2^ |
| 6.5 |  | 1.0 ± 0.00 | (1.1 ± 0.5)×10^-1^ |
| 3.5 | 19 | 0.59 ± 0.06 | (1.0 ± 0.1)×10^-2^ |
| 4.0 |  | 0.84 ± 0.06 | (3.2 ± 0.7)×10^-2^ |
| 4.5 |  | 1.0 ± 0.00 | (0.9 ± 0.2)×10^-1^ |

**S2 Table. The tension dependent probability of pore formation and the rate constant of rupture of DOPG/DOPC (40/60)-GUVs under different osmolarities.**

| Electric tension  *σ*_c_ (mN/m) | Osmolarity difference Δ*C*^0^ (mOsm/L) | Probability of pore formation  *P*_pore_ (60 s) | Rate constant of rupture  *k*_p_ (s^-1^) |
| --- | --- | --- | --- |
| 5.0 | 0 | 0.48 ± 0.04 | (0.9 ± 0.02)×10^-2^ |
| 6.0 |  | 0.89 ± 0.05 | (4.0 ± 0.5)×10^-2^ |
| 7.0 |  | 1.0 ± 0.00 | (1.4 ± 0.1)×10^-1^ |
| 3.0 | 13 | 0.51 ± 0.08 | (0.8 ± 0.2)×10^-2^ |
| 4.0 |  | 0.80 ± 0.06 | (4.7 ± 1.1)×10^-2^ |
| 5.0 |  | 1.0 ± 0.00 | (1.4 ± 0.4)×10^-1^ |
| 2.0 | 17 | 0.57 ± 0.09 | (0.9 ± 0.1)×10^-2^ |
| 2.5 |  | 0.82 ± 0.05 | (3.7 ± 0.5)×10^-2^ |
| 3.0 |  | 1.0 ± 0.00 | (1.1 ± 0.5)×10^-1^ |
